# Prescribing antibiotics when the stakes are higher — do GPs prescribe less when patients are pregnant? A retrospective observational study

**DOI:** 10.3399/bjgpopen18X101505

**Published:** 2018-05-02

**Authors:** Guro Haugen Fossum, Svein Gjelstad, Kari J Kværner, Morten Lindbaek

**Affiliations:** 1 PhD Student, Department of General Practice, Antibiotic Center for Primary Care, Institute of Health and Society, University of Oslo, Oslo, Norway; 2 Associate Professor, Department of General Practice, Antibiotic Center for Primary Care, Institute of Health and Society, University of Oslo, Oslo, Norway; 3 Professor, C3 Centre for Connected Care, Oslo University Hospital and BI Norwegian Business School, Oslo, Norway; 4 Professor, Department of General Practice, Antibiotic Center for Primary Care, Institute of Health and Society, University of Oslo, Oslo, Norway

**Keywords:** general practice, anti-bacterial agents, pregnancy, physicians’ practice patterns, respiratory tract infections

## Abstract

**Background:**

Most oral antibiotics are prescribed by GPs, and they are therefore the most important influencers with regard to improving antibiotic prescription patterns. Although GPs’ prescription patterns in general are well-studied, little is known about antibiotic prescription patterns in pregnancy.

**Aim:**

To study GPs’ antibiotic prescriptions in respiratory tract infections (RTIs) during pregnancy, and assess differences, if any, between pregnant and non-pregnant patients.

**Design & setting:**

Retrospective observational study combining prescription data from the Norwegian Peer Academic Detailing (Rx-PAD) study database, pregnancy data from the Norwegian birth registry, and pharmacy dispension data from the Norwegian Prescription Database (NorPD).

**Method:**

Records of patient contacts with 458 GPs, between December 2004 and February 2007, were screened for RTI episodes. Similar diagnoses were grouped together, as were similar antibiotics. Episodes were categorised according to whether the patient was pregnant or not, and included women aged 16–46 years. Logistic regression models were used to assess odds ratios (ORs), and calculated relative risks (cRRs) were produced. The authors also adjusted for clustering at various levels.

**Results:**

Overall prescription rate for RTI episodes was 30.8% (*n* = 96 830). The cohort was reduced to include only episodes with women pregnant in the study period (*n* = 18 890). The antibiotic prescription rate in pregnancy was 25.9% versus 34.2% in the time before and after pregnancy (cRR = 0.66 [95% confidence intervals {CI} = 0.68 to 0.81]).

During pregnancy, 83.0% of the antibiotic prescriptions were picked up at a pharmacy, compared to an 86.6% filling rate in non-pregnant patients. The difference was not significant when adjusting for clustering at the patient level.

**Conclusion:**

Norwegian GPs prescribe fewer antibiotics overall when patients are pregnant and, when they do prescribe, choose more narrow spectrum antibiotics for RTIs. This indicates a possible lower target rate for GP prescriptions to females. A low antibiotic dispension rate during pregnancy may represent a discussion topic in the consultation setting, to address possible reasons and avoid under-treatment.

## How this fits in

Factors affecting GPs’ general antibiotic prescription pattern are well-studied, but less is known about antibiotic prescriptions in pregnancy. This study shows that GPs prescribe fewer antibiotics, and choose to prescribe more narrow spectrum antibiotics to women with RTIs when the patient is pregnant. These patients also have a lower filling rate of their antibiotic prescriptions.

## Introduction

Antibiotic resistance is considered a major threat to public health;^1^ the link between antibiotic use and resistance is well-established, both on individual patient and national levels.^2–4^ National studies show variations in prescribing, and also over-treatment for some diagnoses and patient groups.^5–7^ In Norway, sales of antibiotics increased by 19% between 1990 and 2010.^8^ Internationally, a shift towards more broad-spectrum antibiotic treatment seems to be the trend.^9^ GPs are likely to be the most important influencers with regards improving antibiotic prescription patterns, as most antibiotics are prescribed in this setting. Addressing prescribing in the consultation may be one approach, where decision-making includes the individual patient's perspective, as recommended in NICE medicines adherence guidelines.^10–12^ As much as 85% of oral antibiotics are prescribed by GPs;^13^ more than half of these are for RTIs, where antibiotics only have a modest effect.^14,15^

Self-limiting infections, such as uncomplicated RTI, are frequent in pregnancy and antibiotics are commonly prescribed. A prevalence of 5% is found in self-report data on infections during a 7-day period in late pregnancy.^16^ Register-based studies report that as many as 20–49% pregnant women receive antibiotics from pharmacies.^17–22^ National and local guidelines may provide antibiotic treatment recommendations in pregnancy, but this is not the norm. Physicians are commonly left to perform specific searches in medical treatment databases such as the British National Formulary,^23^ and the UK Teratology Information Service's clinical toxicology database, Toxbase. In Norway, national guidelines for antibiotic treatment in primary care provide pregnancy-specific antibiotic recommendations. A conservative approach is the rule: penicillins are the first choice; macrolides, second generation cephalosporins, and lincosamides the second choice.^24^

In Norway, GPs are formal gatekeepers in the general antenatal care program. In other European countries, GPs also play a role in maternity care, as well as addressing common illnesses in pregnancy.^25–27^ Shared decision-making in consultations during pregnancy may challenge the autonomy of the GP. Studies report that patient expectations and hopes for antibiotics, and the clinician’s perceptions of the patient’s demands, are predictors of antibiotic prescribing.^10,28,29^ Adhering to evidence-based guidelines may be difficult, particularly if they are non-specific.^30^ Combining patient-centred care with evidence-based medicine in shared decision-making may need support from clinical guidelines.^31^ However, when high impact guidelines, such as NICE,^32,33^ lack specific information on antibiotic treatment in pregnancy, the GP must show flexibility to balance patient demands and adherence to treatment.^34^ Recent studies show that women perceive antibiotic use in pregnancy as a risk, and that this is an important drive in the decision-making during the consultation.^35,36^

Although GPs' prescription patterns in general are well-studied, little is known about antibiotic prescription patterns in pregnancy. Most studies report based on pharmacy prescription dispensions. One UK GP database study found prescription differences, both in choice of antibiotics and indication for uses during pregnancy.^37^ A Norwegian pregnancy study found that, in the period 3 months prior to pregnancy to 3 months post-partum, 44% of women picked up ≥1 anti-infective medication prescription.^19^

To target possible over-prescribing or inappropriate prescribing, information on GPs' prescription patterns during pregnancy is needed. This study's main objective was to explore GPs’ antibiotic prescriptions in RTIs during pregnancy, and assess differences in both prescription rates and choice of antibiotics in pregnant versus non-pregnant patients. Additionally, the authors aimed to study filling rates of antibiotics prescriptions, comparing the same groups of patients.

## Method

### The Rx-PAD study

**Figure 1.**
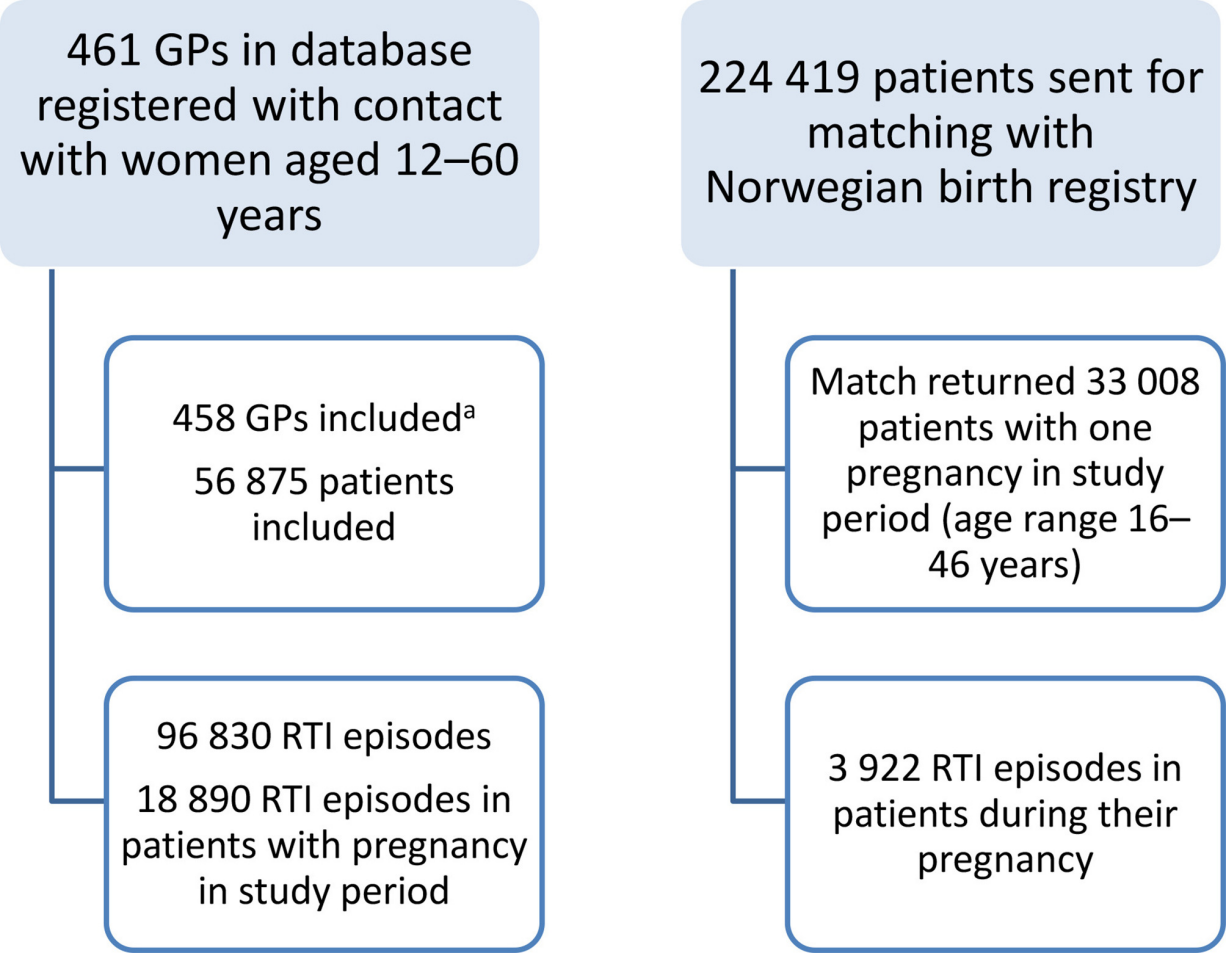
Selection of GPs and patients from the Rx-PAD study database and the Norwegian birth registry in the study period December 2004–February 2007. ^a^GPs with <10 episodes were excluded. RTI = respiratory tract infection. Rx-PAD = Peer Academic Detailing.

Prescription data from GPs was obtained from the Rx-PAD study database, which has been described in detail elsewhere.^38^ This was a cluster randomised educational intervention for GPs aimed at improving the quality of prescribing antibiotics for RTIs.^39^ All GP participants who had consultative contacts with women aged 12–60 years (in order to include all pregnancies), from December 2004 to February 2007, were included in the study; 461 GPs in total. Women without a valid Norwegian birth number and GPs with <10 contacts were excluded. The contacts were later reduced to RTI episodes; all contacts with an individual patient with the same diagnosis in the ICPC-2^40^ within 4 weeks were counted as one RTI episode. Diagnostic ICPC-2 codes reflecting similar illnesses were grouped together.^39^ The antibiotic prescriptions were also classified into antibiotic groups, as described in previous studies.^5,7,39^ Data selection is described in Figure 1.

The first prescription in each RTI episode was linked with the prescription registered at a pharmacy according to NorPD.^41^ The final database provides both prescription and dispension data.

### The medical birth registry

Pregnancy data were obtained from the Norwegian birth registry.^42^ The registry contains data on the expected term of birth and the date of the last menstrual period before pregnancy. Smoking data and data on chronic illnesses were also included from this database. Pregnancy data were linked with the prescription and pharmacy dispension database. The length of the pregnancy was calculated based on the last period and the birth date. Using these dates, all episodes of RTIs at the GP's office occurring during pregnancy were labelled as 'pregnant = 1'. This gave a sub-cohort of all women with a pregnancy in the study period. (Figure 1)

### Analysis

The statistical analyses were performed using the software Stata (version 13.1). A logistic regression model was applied, adjusting for clustering. Analyses were primarily done of the cohort of women with a pregnancy in the study period (18 890 RTI episodes). In the logistic regression model, both the ORs and the cRRs are adjusted for GP factors: urban practice, group practice, sex, age, years since authorisation, number of listed patients, consultations per year, and if they took part in an ongoing intervention study on antibiotic prescriptions. Additionally, looking at the proportion of non-penicillin V prescriptions, the antibiotic prescription rate of the GP was also adjusted for. The patient factors adjusted for are: age, diagnosis, smoking status during pregnancy, and if the patients were registered with asthma on their pregnancy personal health record. The level of clustering adjusted for depended on the number of observations within each cluster. Random intercept was used to adjust for groups of GPs working together, the ID of the GP, in some analyses the patient ID, and according to diagnosis. The main outcome, antibiotic prescriptions, was expected to be frequent. The OR might therefore reflect an overestimated effect, and thus relative risk was calculated from the adjusted odds ratio (aOR) according to Zhang *et al*.^43^

## Results

The cohort included 458 GPs, most of whom were male (68.3%), specialists (86.0%), and working in a group practice (92.4%). Overall prescription rate for RTI episodes was 30.8%, with a mean prescription rate of 29.8 per 100 RTI episodes (Table 1). Based on contacts with 56 875 separate women aged 16–46 years during December 2004–February 2007, 96 830 RTI episodes were registered.

**Table 1.** Characteristics of GPs (*n* = 458)

Characteristic	*n* (%)
**Sex**	
Female	145 (31.7)
Male	313 (68.3)
**Practice location**	
Urban	247 (53.9)
Rural	211 (46.1)
**Practice type**	
Group	423 (92.4)
Single	35 (7.6)
**GP specialist**	
Yes	394 (86)
No	64 (14)
**Mean age, years (SD)**	49.7 (8.1)
**Years since authorisation, mean (SD)**	19.8 (8.7)
**Listed patients^a^, mean (SD)**	1341 (385)
**Patient consultations per year, mean (SD)**	2886 (996)
**Antibiotic prescription rate^b^, mean (SD)**	29.8 (10.9)

^a^Includes only GPs with listed patients (*n* = 449). ^b^Per 100 respiratory tract infection episodes.

The antibiotic prescription rate for RTI episodes among the 11 605 patients with a pregnancy in the study period was 32.5% (*N* = 18 890). The number of RTI episodes occurring during an ongoing pregnancy was 3922 (20.8%).

Most pregnant patients experienced only one episode during the study. The majority of both pregnant and non-pregnant women had 1–3 episodes registered (93.6% versus 91.8%).

The antibiotic prescription rate was lower in pregnancy compared to that in non-pregnant patients (25.9% versus 34.2%; cRR = 0.66 [95% CI = 0.68 to 0.81]). The proportion of non-penicillin V prescriptions was significantly higher among non-pregnant patients (44.7% versus 35.9%; cRR = 0.67 [95% CI = 0.59 to 0.77]) (Table 2). Antibiotic prescription rates were found to vary by ICPC-2 diagnosis, patient's age, GP’s age, and GP’s consultation rates. The proportion of penicillin V prescriptions was influenced by ICPC-2 diagnosis, a patient history of asthma, age, GP’s prescription rate, and whether the GP was part of an antibiotic prescription intervention study.

**Table 2.** Antibiotic prescriptions in respiratory tract infection episodes, showing rates for pregnant and non-pregnant patients, and proportion of non-penicillin V prescriptions for the same groups

		*n* (%)	aOR	cRR (95% CI)
Patients with a pregnancy in study period (*n* = 18 890)				
Antibiotic prescription	Control	5116 (34.2)	Ref	
	Pregnant	1015 (25.9)	0.66	0.74 (0.68 to 0.81)
Non-penicillin V prescriptions	Control	2285 (44.7)	Ref	
	Pregnant	364 (35.9)	0.53	0.67 (0.59 to 0.77)

aOR = adjusted odds ratio. 95% CI = 95% confidence interval. cRR = calculated relative risk. Ref = reference.

**Figure 2.**
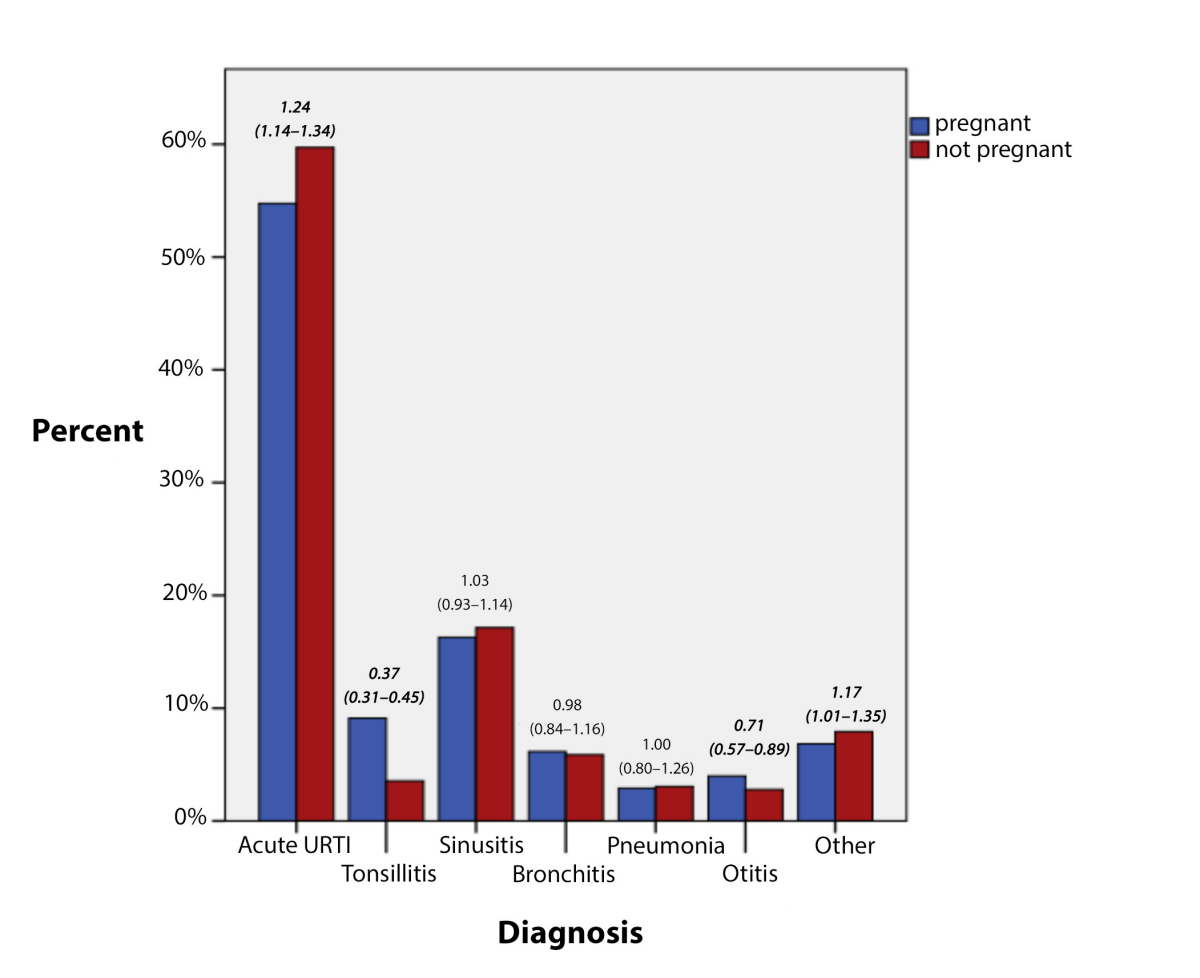
Diagnosis of respiratory tract infection episodes in study period, showing adjusted odds ratio for 'pregnant' and 95% confidence intervals (significant difference in bold and italic, *P*<0.05). Diagnoses based on ICPC-2: acute upper respiratory tract infections, URTI (R01–05, 07–29, 74, and 80), tonsillitis (R72 and 76), sinusitis (R75), bronchitis (R78), pneumonia (R81), otitis (H01, 71, 72, and 74), and other respiratory tract infections (R71, 77, 82, and 83). *N* = 18 890 episodes.

The RTI episodes were analysed by diagnosis and by the GPs’ choice of antibiotic. The distribution of diagnoses in Figure 2 shows that 'acute URTIs (upper respiratory tract infections)' (59.7% versus 54.8%; aOR = 1.24 [95% CI = 1.14 to 1.34]) and 'other' (7.9% versus 6.8%; aOR = 1.17 [95% CI = 1.01 to 1.35]) were slightly more prevalent, and that 'tonsillitis' (3.5% versus 9.1%; aOR = 0.37 [95% CI = 0.31 to 0.45]) and 'otitis' (2.8% versus 4.0%; aOR = 0.71 [95% CI = 0.57 to 0.89]) were less frequent in the pregnant patients. No difference was found for the diagnoses 'sinusitis', 'bronchitis', and 'pneumonia'.

**Figure 3.**
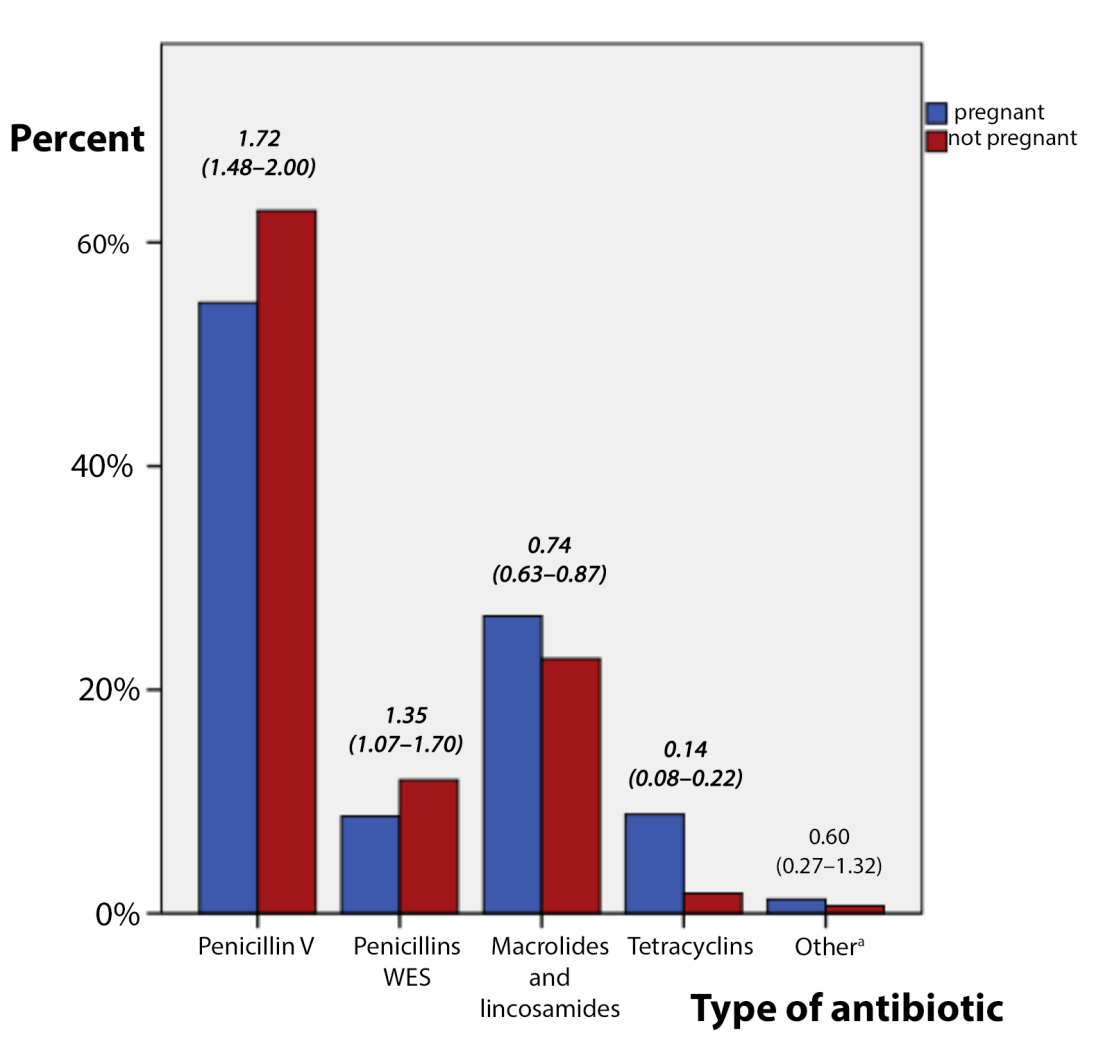
Antibiotic prescriptions for URTIs in patients who were pregnant during study period showing adjusted odds ratio for 'pregnant' and 95% confidence intervals, (significant difference in bold and italic, *P*<0.05). *N* = 18 890 episodes. ^a^Ciprofloxacin and cephalosporins. Penicillins WES = penicillins with extended spectrum.

Penicillin V prescriptions were more frequent when patients were pregnant compared to non-pregnant (62.8% versus 54.6%; aOR = 1.72 [95% CI = 1.48 to 2.00]). Broad-spectrum penicillin prescriptions were also more frequent among pregnant patients, while macrolides, lincosamides, and tetracyclins prescriptions were fewer. (Figure 3)

The mean number of contacts with an antibiotic prescription per individual patient was 1.27. Antibiotic dispension by pharmacy is based on all contacts resulting in an antibiotic prescription for an RTI by the GP with women who were pregnant during the study period; 31 265 in total. During pregnancy, 83.0% of the prescriptions were picked up; the corresponding figure for the time period before and after pregnancy was 86.6% (Table 3). Pregnancy affected the filling rate (aOR = 0.79; cRR = 0.97 [95% CI = 0.94 to 0.99]), but failed to remain significant when adjusting for clustering at the patient level. Diagnoses of tonsillitis, bronchitis, and pneumonia were factors found to influence filling rates. Older age was associated with increased prescription filling rates. A prescription of an antibiotic in the category 'penicillins with extended spectrum' was less likely to be picked up (aOR = 0.82 [95% CI = 0.73 to 0.93]). Clustering at both GP and individual patient level affected the results.

**Table 3.** Antibiotic prescriptions showing rates of dispension at pharmacy (*N* = 31 265 RTI contacts with GP resulting in a prescription of an antibiotic)

		*n* (%)	aOR	RR^a ^(95% CI)
Prescription dispension	Control	26 053 (86.6)	Ref	0.97 (0.94 to 0.99)
	Pregnant	974 (83.0)	0.79	
Prescription dispension, individual^b^	Control	26 053 (86.6)	Ref	0.96 (0.88 to 1.02)
	Pregnant	974 (83.0)	0.77	

^a^Calculated from odds ratio. ^b^The second row shows the rates when random intercept clustering at patient level is also adjusted for.

aOR = adjusted odds ratio. 95% CI = 95% confidence intervals. Ref = reference. RR = relative risk.

## Discussion

### Summary

This study's main findings show that Norwegian GPs prescribe antibiotics less frequently during pregnancy, and that more narrow spectrum antibiotics are prescribed for these patients compared to non-pregnant women. No significant differences were found for prescription filling rates from pharmacies between the pregnant and non-pregnant patients, but rates were generally low at <90%.

### Strengths and limitations

The total size of the database and the large number of RTI episodes are a major strength of this study. This allows for diversity in both patients and GPs, reflecting the population from non-selective recruitment.

Registries like the birth registry and NorPD provide complete data from the entire country.

Healthcare-seeking behaviours were similar for the groups of pregnant and non-pregnant patients. One might assume that pregnant patients see their GP more, thus giving a lower prescription rate. However, these results indicate the same behaviour, with 62.0% of non-pregnant and 64.0% of pregnant patients having only one episode of RTI contact with their GP.

It can be argued that prescription data from 2004–2007 are somewhat old. Newer data show a shift towards more antibiotic consumption in Norway up to 2012, then less consumption up to 2015, giving a present consumption at the same level as in 2006.^13^ Data from the UK show a similar prescription rate for common RTIs between 2004 and 2011.^44^ Also, this study’s aim was to look at different prescribing patterns towards pregnant and non-pregnant patients, and the authors have no reason to believe that this has changed.

The RTI episodes do not take into account patients seeking other GPs out of hours, or using out-patient emergency departments; some patients may therefore have had more contacts than were included in this study's database. However, it is the GPs' prescription patterns that are being examined in these analyses, and the study still includes all the information on the participating doctors.

The RTI consultation may happen before both patient and GP know about the pregnancy. However, <10% of episodes with a pregnant patient are registered before 3 weeks of pregnancy, and after that most women will know if they are pregnant.

### Comparison with existing literature

Previous studies from Norway have shown prescription rates for RTIs in primary care at about 33%.^7^ This study adds to this when looking at the control group. The present authors found no studies addressing GPs’ prescription rate for RTIs in pregnancy, only register-based studies on filled prescriptions from pharmacies. Petersen *et al* showed that 34% of British women were prescribed ≥1 antibiotic during pregnancy in 2007, rising from 30% in 2002–2003.^37^ The corresponding figure from a recent Danish study was 41.5%.^45^ Amann *et al* analysed antibiotics dispensed to pregnant women in Germany in 2000–2001, and showed a shift to relatively safe and reduced antibiotic drug use during pregnancy.^17^ A study from Italy showed that antibiotics were prescribed less commonly during pregnancy than in the same time period the year before.^46^

The GPs in the study tend to choose narrow spectrum antibiotics when the patient is pregnant. This may be related to patient choice and shared decision-making, as patients are found to be restrictive and unsure about medication use during pregnancy.^47^ Narrow spectrum penicillin is also considered the drug of choice when prescribing to pregnant women, according to guidelines.^24^ Petersen *et al* showed a similar shift in pregnancy towards fewer macrolides, and an increase in broad spectrum penicillins, but not the same increase in the choice of narrow spectrum penicillin found in the present study.^37^ The unique situation in Norway, where GPs have a high rate of penicillin V prescriptions (about 50%) in RTI episodes, must be taken into account in the interpretation of the results.^39^

Adequate treatment is essential, and although the proportion of penicillin V prescriptions for RTIs in the UK is lower than in Norway, it is not associated with treatment failure.^48^

This study did not find the prescription of contraindicated antibiotics to be a major problem, as only one prescription of a tetracycline after 15 weeks of pregnancy was registered. Recent Norwegian guidelines recommend that macrolides are used only on extraordinary indications after 28 weeks of pregnancy.^24^ At the time of the study, Norwegian guidelines did not question the use of macrolide antibiotics in pregnancy; this study found them frequently to be a second choice.^49^ Macrolides are also one of the antibiotics found in a recent study to be overrepresented in prescriptions to women aged 16–54 years.^50^

When analysing the distribution of diagnoses according to ICPC-2, a shift was found towards more symptom diagnoses, such as 'acute URTIs' and 'other’, from more specific disease diagnoses like ‘tonsillitis’ and ‘otitis’. The authors suspect that the choice of diagnosis is influenced by the GP’s decision of whether or not to prescribe antibiotics. This is in accordance with a Dutch study, in which Van Duijn *et al* found that GPs in Holland tend to diagnose more patients with infections than with symptoms when they prescribe an antibiotic.^51^ Tonsillitis has earlier been addressed as one of the problem diagnoses when using ICPC-2 in a Norwegian GP setting.^52^ Other studies have also pointed out how completeness and correctness of data entry may rely on the individual GPs.^53^

The filling rates of antibiotic prescriptions from pharmacies in pregnancy were found to be low compared to the rates in non-pregnancy. A previous Norwegian study, published in 2013, did not show that either sex or age groups between 19 and 44 years were factors affecting filling rates.^54^ The baseline overall filling rate of 92.1% in that study differs from that found in pregnant women in the present study, which is 83.0%.^54^ In a study from the US, as many as 26% of patients did not fill their prescriptions of antibiotics from the emergency department within the first 24 hours after discharge.^55^ Medication non-adherence in pregnancy is considered common.^56^ Olesen *et al* found that Danish pregnant women had an adherence to antibiotics of only 52%.^57^ Recent studies show that pregnant women deliberately avoid the use of certain medicines during pregnancy.^36^ This, together with a possible increased use of delayed prescriptions in pregnancy may have contributed to the low filling rates.

### Implications for practice

Norwegian GPs seem to adhere to national guidelines to a greater degree when prescribing antibiotics to pregnant women than to the general population, with a lower prescription rate and fewer broad spectrum antibiotics. GPs prescribe antibiotics when they think patients expect them.^58^ The patient’s beliefs and perception of risk is an important factor for healthcare workers to consider, especially in pregnancy.^35,36^ Involving the patient before a decision is made and asking about their expectations for antibiotics reduces prescriptions in acute RTIs.^59,60^ When the risk is high, both patients and GPs seek to avoid harming the fetus, and the guidelines are more strictly followed.

The low prescription rate and the frequent choice of narrow spectrum antibiotics by GPs in these patients may represent their lowest target and a possible aim for all young women with RTIs. The low filling rate of antibiotic prescriptions in pregnant patients may also indicate a general scepticism towards prescription drugs in this group. GPs need to consider talking to their patients about this to avoid possible under-treatment.
